# Hydrojet-based delivery of footprint-free iPSC-derived cardiomyocytes into porcine myocardium

**DOI:** 10.1038/s41598-020-73693-x

**Published:** 2020-10-08

**Authors:** Marbod Weber, Andreas Fech, Luise Jäger, Heidrun Steinle, Louisa Bühler, Regine Mariette Perl, Petros Martirosian, Roman Mehling, Dominik Sonanini, Wilhelm K. Aicher, Konstantin Nikolaou, Christian Schlensak, Markus D. Enderle, Hans Peter Wendel, Walter Linzenbold, Meltem Avci-Adali

**Affiliations:** 1 Department of Thoracic and Cardiovascular Surgery, University Hospital Tuebingen, Calwerstraße 7/1, 72076 Tuebingen, Germany; 2 Erbe Elektromedizin Tuebingen, Waldhoernlestr. 17, 72072 Tuebingen, Germany; 3 Diagnostic and Interventional Radiology, University Hospital Tuebingen, Hoppe-Seyler-Strasse 3, 72076 Tuebingen, Germany; 4 Department of Preclinical Imaging and Radiopharmacy, Werner Siemens Imaging Center, Eberhard Karls University, Roentgenweg 13, 72076 Tuebingen, Germany; 5 Department of Urology, ZMF, University Hospital Tuebingen, Waldhoernlestr. 22, 72072 Tuebingen, Germany

**Keywords:** Heart failure, Cardiac regeneration

## Abstract

The reprogramming of patient´s somatic cells into induced pluripotent stem cells (iPSCs) and the consecutive differentiation into cardiomyocytes enables new options for the treatment of infarcted myocardium. In this study, the applicability of a hydrojet-based method to deliver footprint-free iPSC-derived cardiomyocytes into the myocardium was analyzed. A new hydrojet system enabling a rapid and accurate change between high tissue penetration pressures and low cell injection pressures was developed. Iron oxide-coated microparticles were ex vivo injected into porcine hearts to establish the application parameters and the distribution was analyzed using magnetic resonance imaging. The influence of different hydrojet pressure settings on the viability of cardiomyocytes was analyzed. Subsequently, cardiomyocytes were delivered into the porcine myocardium and analyzed by an in vivo imaging system. The delivery of microparticles or cardiomyocytes into porcine myocardium resulted in a widespread three-dimensional distribution. In vitro, 7 days post-injection, only cardiomyocytes applied with a hydrojet pressure setting of E20 (79.57 ± 1.44%) showed a significantly reduced cell viability in comparison to the cells applied with 27G needle (98.35 ± 5.15%). Furthermore, significantly less undesired distribution of the cells via blood vessels was detected compared to 27G needle injection. This study demonstrated the applicability of the hydrojet-based method for the intramyocardial delivery of iPSC-derived cardiomyocytes. The efficient delivery of cardiomyocytes into infarcted myocardium could significantly improve the regeneration.

## Introduction

Cardiovascular diseases (CVDs) are the main cause of death with over 17 million deaths globally per year, which represents 30% of all deaths, and it is expected to reach 23.6 million by 2030^1^. CVDs include disorders that can affect the heart and the blood vessels, such as angina, myocardial infarction, and peripheral artery disease. The death of cardiomyocytes following myocardial infarction induces a reaction cascade leading to cell death within hours after coronary artery occlusion^2^ and ending with collagen matrix production and scar formation^3^.

Unfortunately, cardiomyocytes have an extremely low renewal capacity with approximately 1% per year at the age of 20, declining to less than 0.5% per year in elderly individuals^4^. Due to this very limited regeneration capacity of the adult human hearts, lost contractile cells are replaced by a fibrotic scar produced by fibroblasts and myofibroblasts. This leads to the remodeling of the myocardium including thickening (hypertrophy) and stiffening (fibrosis) of the left ventricular wall^5^ and ultimately results in impaired cardiac function.

To date, various cell-based approaches^6^ were clinically tested to promote cardiac regeneration by integration, differentiation, and proliferation of implanted cells, such as the application of mesenchymal stromal cells (MSCs) isolated from bone marrow^7^ or adipose tissue-derived stem cells^8^, skeletal myoblasts^9^, circulating progenitor cells^10^, and cardiac stem cells^11^. However, recent data suggest that the clinically observed benefit associated with the injection of bone marrow-derived cells is primarily due to the release of paracrine factors^12,13^. But there is a certain risk for spontaneous osteogenic differentiation of such cells after the in vivo application. For instance, intramyocardial calcification was reported after transplantation of unpurified bone marrow cells into the infarcted myocardium of rats^14^. In another study, encapsulated calcified or ossified structures were also found after the injection of MSCs into infarcted mice hearts^15^.

The discovery of the reprogrammability of somatic cells in induced pluripotent stem cells (iPSCs)^16,17^ opened up new possibilities for regenerative therapies in general. These cells have the potential to differentiate into a variety of cell types of the body and the unlimited proliferation capacity of iPSCs could particularly allow the generation of large numbers of autologous cardiomyocytes for the repair of infarcted myocardium. Since the first generation of iPSCs from fibroblasts by using retroviral vectors^16,17^, other integrative vectors such as lentiviral vectors^18^, plasmids^19^, or piggyBac transposon-based delivery systems^20^ were also used to reprogram somatic cells. New strategies are focusing on non-integrative methods, such as the use of adenoviral^21^, Sendai^22^, and episomal vectors^23^, mRNA^24^, or proteins^25^ to obtain clinically applicable terminally differentiated cells from iPSCs. In a recent study, we have shown that by using self-replicating RNA, iPSCs could be generated from human urine-derived renal epithelial cells and beating cardiomyocytes were obtained from these iPSCs^26^. This method allows the non-invasive and simple collection of patient´s somatic cells for reprogramming and the footprint-free generation of iPSCs for subsequent differentiation into cardiomyocytes, which can be used to repair infarcted myocardium.

Regarding cell delivery strategy and functional outcome, an inconclusive view arises from the literature. For example, the incorporation of endothelial and smooth muscle cells into patches increased the resistance of cardiomyocytes to hypoxic injury as well as the engraftment of transplanted cardiomyocytes^27^ and the implantation of such fibrin patches enhanced left ventricular function in a porcine myocardial infarction model^28^. However, Gerbin and colleagues reported the formation of scar tissue that physically separated the epicardial patch from the host myocardium^29^. In contrast, the injection of cardiomyocytes directly into the myocardium resulted in electrical integration of implanted cells^29,30^. However, covering a large area of myocardial infarction may require multiple injections, leading to procedural damage to the myocardium^31^ and should, therefore, be avoided. Recently, Jäger et al.^32^ described a hydrojet-based method for the delivery of MSCs into urethral tissue. They demonstrated a better yield of viable cells compared to needle injections with a fast and precise injection of viable next to or into the sphincter muscle. Here, we aimed to evaluate this new hydrojet concept for the delivery of cardiomyocytes derived from footprint-free generated iPSCs into porcine myocardium. The application was compared to the needle-based application of iPSC-derived cardiomyocytes.

## Material and methods

### Ethics statement

Renal epithelial cells were isolated from the urine of healthy donors, which gave written informed consent to participate. The study was approved by the Ethics Committee of the Medical Faculty of the University of Tuebingen (911/2018BO2). All experiments were performed in accordance with relevant guidelines and regulations. Since no living animals were used in this study, ethical approval for animal testing was not required. Hearts of German Landrace pigs were purchased from a regional butcher´s shop (Faerber, Balingen, Germany).

### Cultivation of footprint-free iPSCs from urine-derived renal epithelial cells

Footprint-free iPSCs were generated as previously described in our recent study^26^ by seeding of 5 × 10^4^ renal epithelial cells obtained from 100–200 ml urine of healthy donors per well of a 12-well plate coated with 0.1% gelatin. The reprogramming was performed by transfection with 0.5 µg self-replicating RNA (VEE-OKSiM-GFP RNA). The generated iPSC colonies were detached and seeded onto 0.5 µg/cm^2^ vitronectin-coated (Thermo Fisher Scientific, Waltham, USA) tissue culture flasks. The cells were cultivated in Essential 8 (E8) medium (Thermo Fisher Scientific) at 37 °C and 5% CO_2_ with daily medium changes and passaged every 4–6 days. After reaching confluence, iPSCs were washed with Dulbecco’s phosphate-buffered saline (DPBS, Thermo Fisher Scientific) and detached by 5 min incubation with DPBS containing 0.5 mM ethylenediaminetetraacetic acid (EDTA, Sigma-Aldrich, St. Louis, USA). After detachment, the EDTA solution was aspirated and the cells were rinsed with E8 medium. 2 × 10^5^ cells were seeded per well of vitronectin-coated 6-well plates in E8 medium containing 10 µg/ml ROCK inhibitor Y-27632 (Enzo Life Sciences, Lausen, Switzerland).

### Generation of iPSC-derived cardiomyocytes

To generate cardiomyocytes, 2 × 10^5^ iPSCs were resuspended in E8 medium containing 10 µg Y-27632 and seeded per well of vitronectin-coated six-well plates. For the differentiation, PSC cardiomyocyte differentiation kit (Thermo Fisher Scientific) was used according to manufacturer’s instructions with small modifications. On day 0, the medium was changed to cardiomyocyte differentiation medium A. On day 2, the medium was changed to cardiomyocyte differentiation medium B. On day 4, cardiomyocyte maintenance medium (CMM) was added to the cells and further cultivated until day 10 to 12 with medium changes every other day. The differentiation of iPSCs into cardiomyocytes was determined by flow cytometry and immunofluorescence microscopy using PE-labeled mouse anti-human α-actinin and FITC-labeled anti-human cardiac troponin T antibodies (both from Miltenyi Biotec, Bergisch Gladbach, Germany). A more detailed characterization of the cardiomyocytes generated from footprint-free iPSCs was performed in our recent study^26^.

### Modified ERBEJET^®^2

A modified ERBEJET^®^2 from Erbe Elektromedizin GmbH (Tuebingen, Germany) was used to apply cells and microparticles into the porcine heart muscle. The new hydrojet system^32^ allowed the generation of pressures (E = effects) ranging from E5 to E80 while enabling a rapid and accurate change between tissue penetration pressures and cell injection pressures. First, using a “tissue penetration jet”, 1 ml 0.9% NaCl solution was applied with high pressures E60 or E80 to penetrate the heart tissue. Afterwards, 100 µl cell or microparticle suspension was delivered using an “injection jet” with low pressures (E5, E10, or E20) to distribute the cells or particles within the penetrated myocardium.

### Application of magnetic polystyrene microparticles into porcine hearts

To simulate and predict the distribution of cardiomyocytes after the injection into the myocardium using a 27G needle or the new hydrojet system, magnetic polystyrene microparticles (Sigma-Aldrich; 6.8 × 10^6^ particles/ml) with a similar diameter as cardiomyocytes (10 ± 0.5 µm) were chosen and injected into porcine hearts. Therefore, porcine hearts were rinsed with 0.9% NaCl solution, sealed in plastic bags and warmed in a water bath to 37 °C to simulate physiological body temperature. Afterwards, using the hydrojet system, the microparticles were injected in the transversal plane 2 cm above the apex from two sites at a 90° angle to the left lateral and dorsal side. Tissue penetration pressures of E60 or E80 were applied combined with an injection pressure of E10—expressed as E60/E10 and E80/E10, respectively. Based on a preliminary phantom study (data not shown), two different quantities of microparticles (85,000 or 42,500) were selected for the assessment of the optimal particle concentration in terms of magnetic resonance imaging (MRI) detection of the artifact signal.

For each hydrojet injection, 100 µl 0.9% NaCl with 85,000 or 42,500 microparticles was used. As a reference, microparticles were also injected into heart muscles using a 27G needle. Therefore, the needle was inserted 2 cm above the apex at a 90° angle from two sites 0.5 cm deep into the myocardium. The hearts were positioned in beakers filled with 0.9% NaCl and MRI was performed.

### MRI of porcine hearts

The microparticle injected hearts were scanned on a 3 T MRI system (MAGNETOM Prisma^fit^, Siemens Healthineers, Erlangen, Germany). The body coil was used for homogeneous radio frequency transmission and a 20-channel head coil was utilized for signal receiving. Morphology and other structures of the hearts were assessed by using a proton-density weighted fast spin-echo sequence with TR/TE = 3000/11 ms, echo train length of 10, acquisition bandwidth of 240 Hz/pixel, field-of-view of 128 × 128 mm^2^, slice thickness of 2 mm, 21 slices, 384 × 384 matrix, two acquisitions, and scan time of 3:50 min. To identify depositions of injected microparticles, series of images were acquired using gradient-echo (GRE) sequence with multiple echo times (TEs). The GRE sequence was used as an MRI technique sensitive to the distribution of the Larmor frequency in the immediate vicinity of the magnetic microparticles. Higher amounts of magnetic microparticles result in a reduced effective transversal relaxation time and can be localized as negative contrasted signal voids in the magnitude images. In addition, the magnetic microparticles produce field inhomogeneities, which can be seen as a characteristical dipole field pattern in the GRE phase images. An identifier for the magnetic microparticles in contrast to the tissue with low signal intensity is the enlargement of the size of the signal voids with increasing TE. The orientation, position, thickness, and number of slices in measurements with the GRE sequence were identical to those in the fast spin-echo (FSE) sequence. The imaging parameters were: TR = 42 ms; TEs = 2.65, 6.71, 10.77, 14.83, 18.89, and 22.95 ms; acquisition bandwidth = 650 Hz/Pixel; flip angle = 25°; field-of-view = 128 × 128 mm^2^; matrix = 256 × 256; acquisition = 2; and a scan time of 5:06 min.

### Analysis of microparticle distribution in porcine hearts

To compare the distribution of microparticles applied into myocardial tissue using hydrojet or 27G needle, DICOM (Digital Imaging and Communications in Medicine) data received from MRI were analyzed using 3D Slicer software (3D Slicer software, version 4.10.2). All sections were retraced with the segmentation function, reconstructed to a three-dimensional (3D) shape considering the layer thickness (2 mm) and layer distance (0.2 mm), and displayed with a smoothing factor of 0.5. Subsequently, the dark particle spots were reconstructed in the same way and the volumes were determined using the segmentation statistics function.

### Analysis of the viability of cardiomyocytes after the application with the hydrojet system

Cardiomyocytes obtained 10–12 days after the differentiation of iPSCs were washed with 1 ml DPBS per well and detached using 1 ml TrypLE (Thermo Fisher Scientific) for 10 min. Afterwards, 1 ml trypsin neutralization solution (TNS, PromoCell, Heidelberg, Germany) was added per well of the six-well plate. Cells were centrifuged at 200 × g for 5 min and washed once with 5 ml DPBS. Afterwards, cardiomyocytes were resuspended in CMM with a final concentration of 1 × 10^7^ cells/ml. To analyze the impact of the injection jet pressure on the viability of cardiomyocytes, 100 µl cell suspension containing 1 × 10^6^ cardiomyocytes was injected and collected in 15 ml tubes filled with 2 ml prewarmed CMM. Injection pressures of E5, E10, and E20 were investigated. The same procedure was also performed by manual injection of 100 µl cell suspension with a 27G needle syringe. Cells were counted in a Neubauer chamber and the viability was determined using trypan blue (Thermo Fisher Scientific). In an additional experiment, the same injection procedures were repeated and the cardiomyocytes were centrifuged at 200 × g for 5 min, resuspended, and seeded into vitronectin coated 48-well plates in CMM. The cell viability was measured after 24 h and 7 days using PrestoBlue assay. Therefore, the medium was replaced by 1 ml PrestoBlue solution (Thermo Fisher Scientific), diluted 1:10 in CMM, and cells were incubated for 90 min. The metabolized solution was then analyzed in triplicates using a fluorescence microplate reader (Mithras LB 940, Berthold Technologies, Bad Wildbad, Germany). Additionally, the cells were stained using calcein AM (Thermo Fisher Scientific) and analyzed using Axiovert135 microscope and AxioVision 4.8.2 software (Carl Zeiss).

### Labeling of the cardiomyocytes with near-infrared fluorescent dye and application into porcine hearts

For the staining, 1 × 10^7^ cardiomyocytes were resuspended in 1 ml DPBS and incubated for 20 min with 300 µM XenoLight DiR fluorescent dye (PerkinElmer, Waltham, MA, USA) dissolved in DMSO. Afterwards, cells were centrifuged at 200 × g for 5 min, washed twice with 5 ml DPBS and resuspended in 1 ml prewarmed CMM medium.

Porcine hearts were rinsed with 0.9% NaCl solution, sealed in plastic bags and warmed up to 37 °C in a water bath to simulate physiological body temperature. Before injection, hearts were cut 5 cm horizontally above the apex and the upper part was removed. This allowed better positioning and observation of the hearts by an vivo imaging system (IVIS Spectrum, Perkin Elmer). Then, 1 × 10^6^ XenoLight DiR fluorescent dye-labeled cardiomyocytes resuspended in 100 µl CMM or 100 µl CMM without cells were injected at a 90° angle 2 cm above the apex into the myocardium either using the new hydrojet system (E60/E10, E80/E10) or a 27 G needle.

### Detection of injected cardiomyocytes using IVIS in porcine heart

After the injection of cardiomyocytes, near-infrared imaging was performed using IVIS. The imaging was performed by placing the apex upwards. Subsequently, the apex was cut sagittally at the injection site into two parts and imaging was performed again. Fluorescence intensity and distribution areas were analyzed with Living Image^®^ software (PerkinElmer). In order to measure the distribution of cells only in the tissue (myocardium), the detected fluorescence of cells distributed via blood vessels was analyzed separately. The fluorescence emission was normalized to photons per second per square centimeter per steradian and expressed as average radiant efficiency [p/s/cm^2^/sr]/[µW/cm^2^].

### Statistical analysis

Data are shown as mean ± standard deviation (SD) or standard error of the mean (SEM). The comparison of the means of normally distributed data was performed by paired t-test or one-way analysis of variance (ANOVA) for repeated measurements followed by Bonferroni’s multiple comparison test. The means of non-normally distributed data were compared using Kruskal–Wallis test followed by Dunn's multiple comparison test. Statistical analyses were performed double-tailed using GraphPad Prism 6.01 (GraphPad Software, La Jolla, CA, USA). Differences of p < 0.05 were considered significant.

## Results

### Analysis of differentiated cardiomyocytes from iPSCs

**Figure 1:**
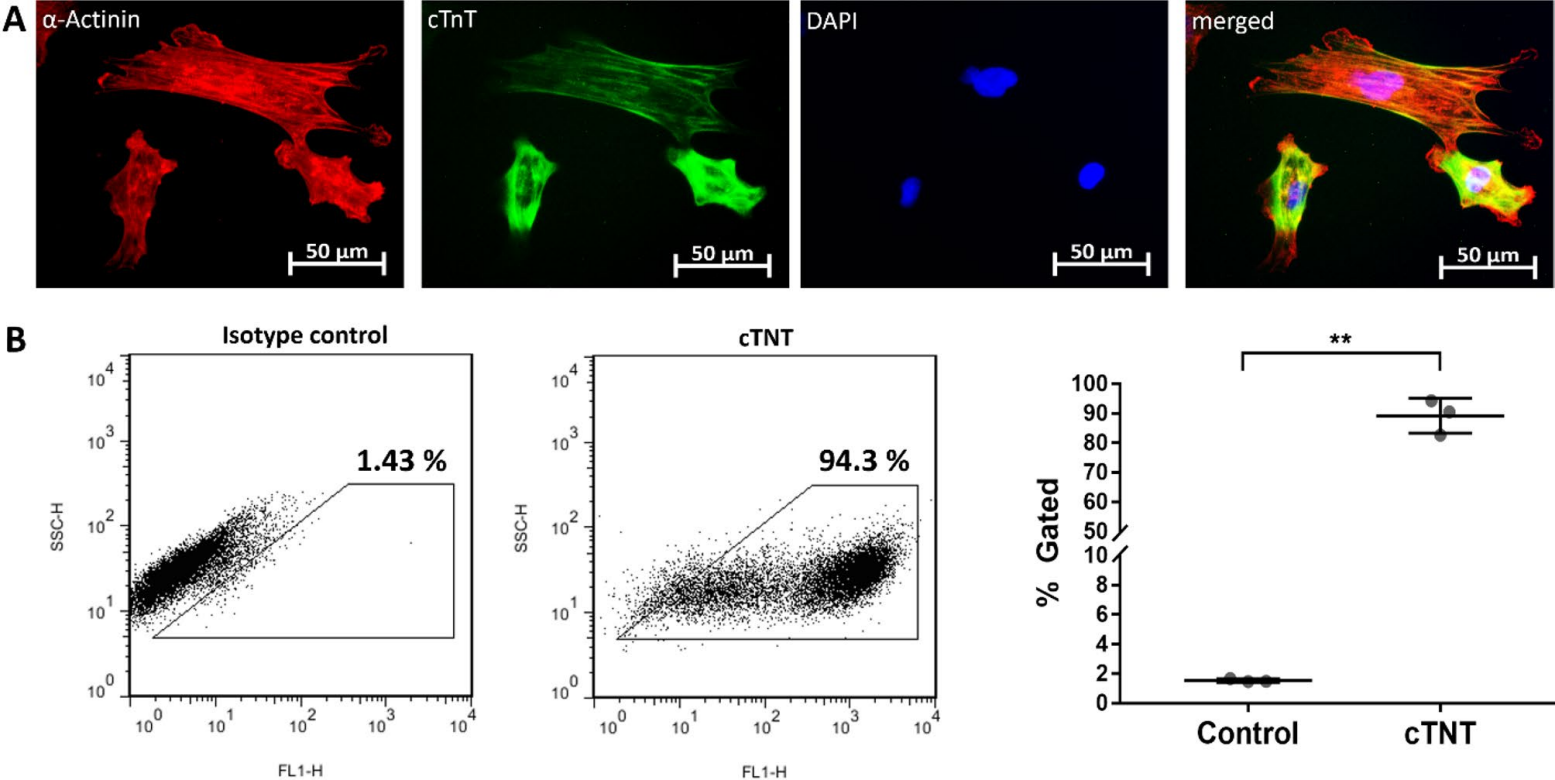
Analysis of cells obtained after cardiac differentiation of iPSCs. **(A)** Fluorescence microscopic images of cTNT and α-actinin positive cells after cardiomyocyte differentiation. Nuclei were stained with DAPI. **(B)** Detection of cTNT positive cells after the cardiac differentiation by flow cytometry. Results are shown as mean ± SD (n = 3). Statistical differences were determined using paired t-test (**p < 0.01).

Using the PSC cardiomyocyte differentiation kit, iPSCs were differentiated within 10–12 days into beating cardiomyocytes. The obtained cells showed the typical elongated rod-like shape of cardiomyocytes and the fluorescence microscopic analyses demonstrated the expression of cTNT as well as α-actinin (Fig. 1A). Flow cytometry analysis revealed that 89.17 ± 5.9% of obtained cells were cTNT positive (Fig. 1B) (p** = 0.0016).

### Particle distribution in porcine hearts

**Figure 2:**
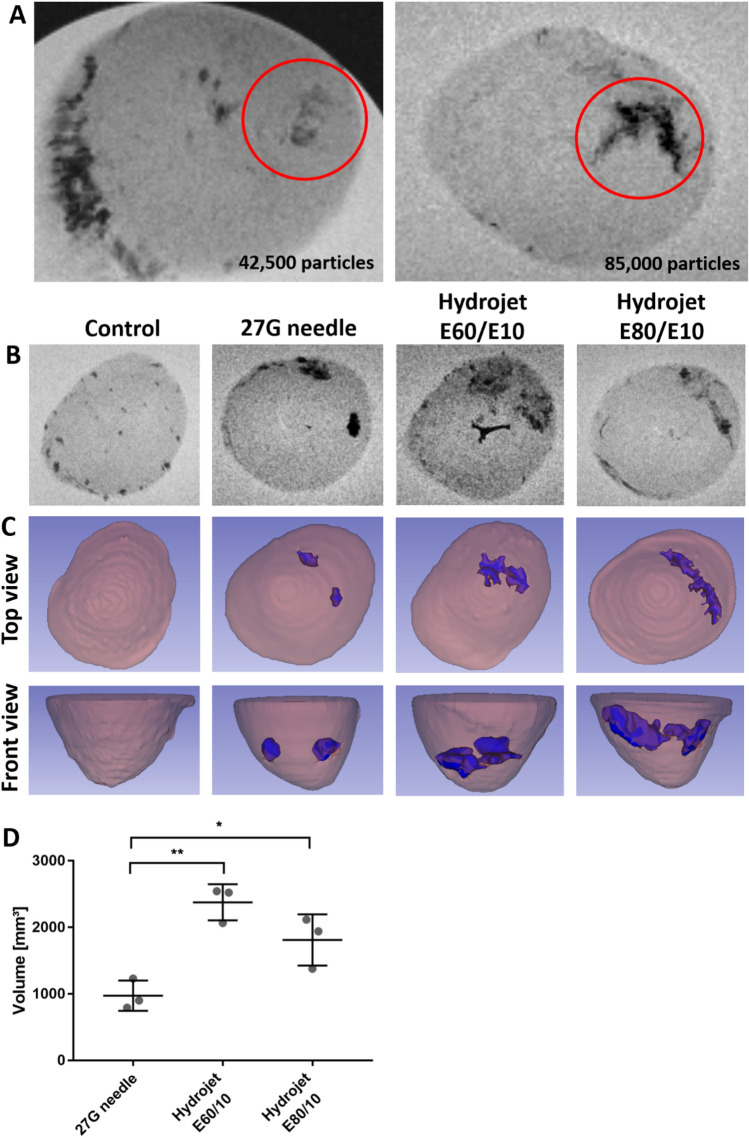
Detection of microparticle distribution in the porcine hearts using magnetic resonance imaging (MRI). **(A)** Determination of detectable microparticle amount in porcine hearts using 45,000 or 85,000 microparticles. A single injection of particles was performed using the new hydrojet system with tissue penetration pressure of E80 and injection pressure of E10 (E80/E10). The microparticles in the heart are highlighted by a red encircled region. (**B)** MRI of microparticles after the application of 85,000 microparticles (two injections) using 27G needle or the hydrojet system with tissue penetration pressures of E80 or E60 and an injection pressure of E10 (E80/E10 or E60/E10). (**C)** 3D reconstruction of particle distribution in porcine hearts using 3D Slicer software. (**D)** Comparison of microparticle distribution volume in porcine hearts. Results are shown as mean ± SD (n = 3). Statistical differences were determined using one-way ANOVA followed by Bonferroni’s multiple comparison test (*p < 0.05; **p < 0.01).

After the application of 85,000 or 42,500 magnetic polystyrene microparticles into porcine hearts using the new hydrojet system and the E80/E10 pressure setting, MRI was performed. The application of 85,000 microparticles led to a stronger artifact signal than 42,500 microparticles (Fig. 2A). Therefore, 85,000 microparticles were applied in further experiments. To compare the distribution of microparticles after the needle- and hydrojet system-based application, microparticles were injected from two sites 2 cm above the apex at a 90° angle to each other and the sagittal plane. After the needle injection, two small localized but strong artifacts were detected in the MRI images near the sites of injection. In contrast, the application of microparticles using the hydrojet system with penetration pressures of E60/E10 and an injection pressure of E10 resulted in a wider distribution of the microparticles in the myocardium (Fig. 2B). However, the higher tissue penetration pressure E80 led to a channel formation, which was clearly visible after the reconstruction of the 3D structure of the heart using 3D Slicer software (Fig. 2C). Compared to the 27G needle injection, both applications with the hydrojet system showed significantly larger distribution volumes [Fig. 2D; E60/E10: 2377 ± 270 mm^3^ (p** = 0.0038); E80/E10: 1811 ± 386 mm^3^ (p* = 0.0439); 27G needle: 975 ± 228 mm^3^].

### Viability and recovery of cardiomyocytes after the application with hydrojet system

**Figure 3:**
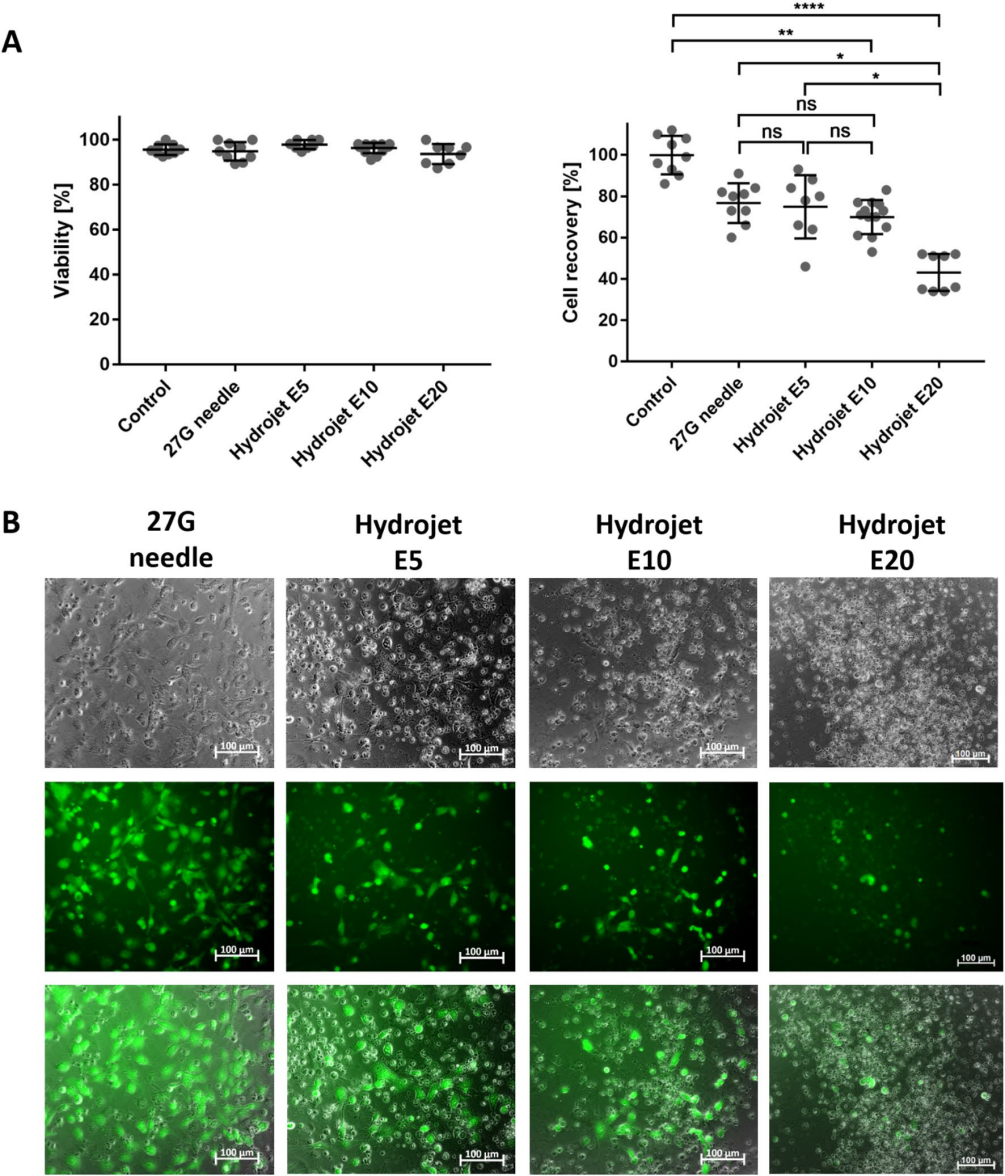
Analysis of the viability and recovery rates of cardiomyocytes immediately after the injection using hydrojet or 27G needle and calcein AM staining of injected cells 24 h after cultivation.** (A)** Determination of cell viability and cell recovery by trypan blue staining and counting of cells immediately after the injection of 1 × 10^6^ cardiomyocytes in CMM using a 27G needle or the hydrojet system with different injection pressure settings (E5, E10, or E20). Results are shown as mean ± SD (control, 27G needle (n = 9), E5 and E20 (n = 8), E10 (n = 15). Statistical differences were determined using one-way ANOVA followed by Bonferroni’s multiple comparison test or Kruskal–Wallis test followed by Dunn's multiple comparison test (*p < 0.05; **p < 0.01; ****p < 0.0001, ns: non-significant). (**B)** Representative images of calcein AM stained cardiomyocytes 24 h after the cultivation of injected cells in cell culture plates for 24 h.

To analyze the recovery and viability of cardiomyocytes, 1 × 10^6^ cells were injected with different pressures, E5, E10, and E20, respectively, as well as 27G needle into CMM. The cell viability and recovery rate were determined using trypan blue staining. No differences in cell viability were detected between the application with 27G needle and hydrojet system (Fig. 3A). The use of 27G needle and hydrojet with a pressure setting of E5 and E10 resulted in an initial cell number loss of 23.3% (27G needle), 25.1% (E5) and 30.1% (E10). However, an injection pressure of E20 led to significantly reduced cell recovery (43.1 ± 9.0%) compared to 27G needle application [76.7 ± 9.6%, (*p = 0.0121)] and the use of injection pressure E5 [74.9 ± 9.6%, (*p = 0.0261)]. These results were also confirmed by performing calcein AM staining 24 h after seeding of recovered cells (Fig. 3B).

**Figure 4:**
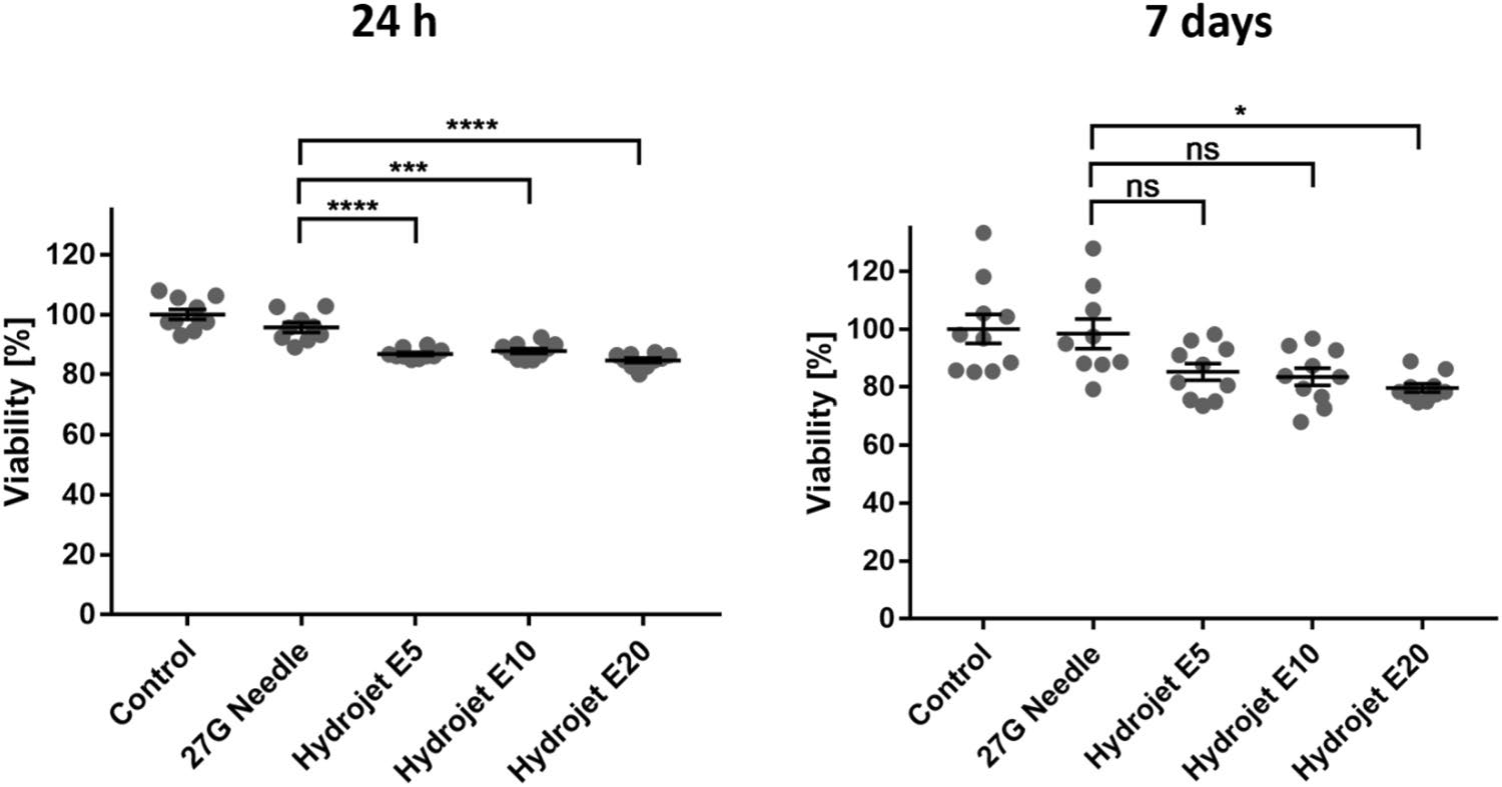
Analysis of the viability of cardiomyocytes 24 h and 7 days after the application of cells. Determination of cell viability using PrestoBlue assay 24 h and 7 days after the seeding of injected cardiomyocytes into cell culture plates. The viability of cardiomyocytes without injection (control) was set to 100% and the viability of cardiomyocytes injected by 27G needle or hydrojet was expressed relative to these cells. Results are shown as mean ± SEM [control, E5, E10, and E20 (n = 10), and 27G needle (n = 9)]. Statistical differences were determined using one-way ANOVA followed by Bonferroni’s multiple comparison test (*p < 0.05, ***p < 0.001, ****p < 0.0001).

Additionally, PrestoBlue assay was performed after 24 h and 7 days of cultivation of cardiomyocytes injected by 27G needle or the hydrojet system with different injection pressure settings (E5, E10, or E20) (Fig. 4). The cells applied by hydrojet technique showed only a slightly reduced cell viability at injection pressure settings of E5 [86.8 ± 0.5% (****p < 0.0001)], E10 [87.82 ± 0.83% (***p = 0.0002)], and at E20 [84.69 ± 0.72% (****p < 0.0001)] compared to the 27G needle injected cardiomyocytes (95.64 ± 1.6%). However, 7 days after the injection, only the cells applied with a pressure setting of E20 showed a significantly reduced cell viability versus the cells applied by 27G needle (27G needle: 98.35 ± 5.15% versus E20: 79.57 ± 1.44%, *p = 0.0102).

### Distribution of applied cardiomyocytes in porcine hearts

**Figure 5:**
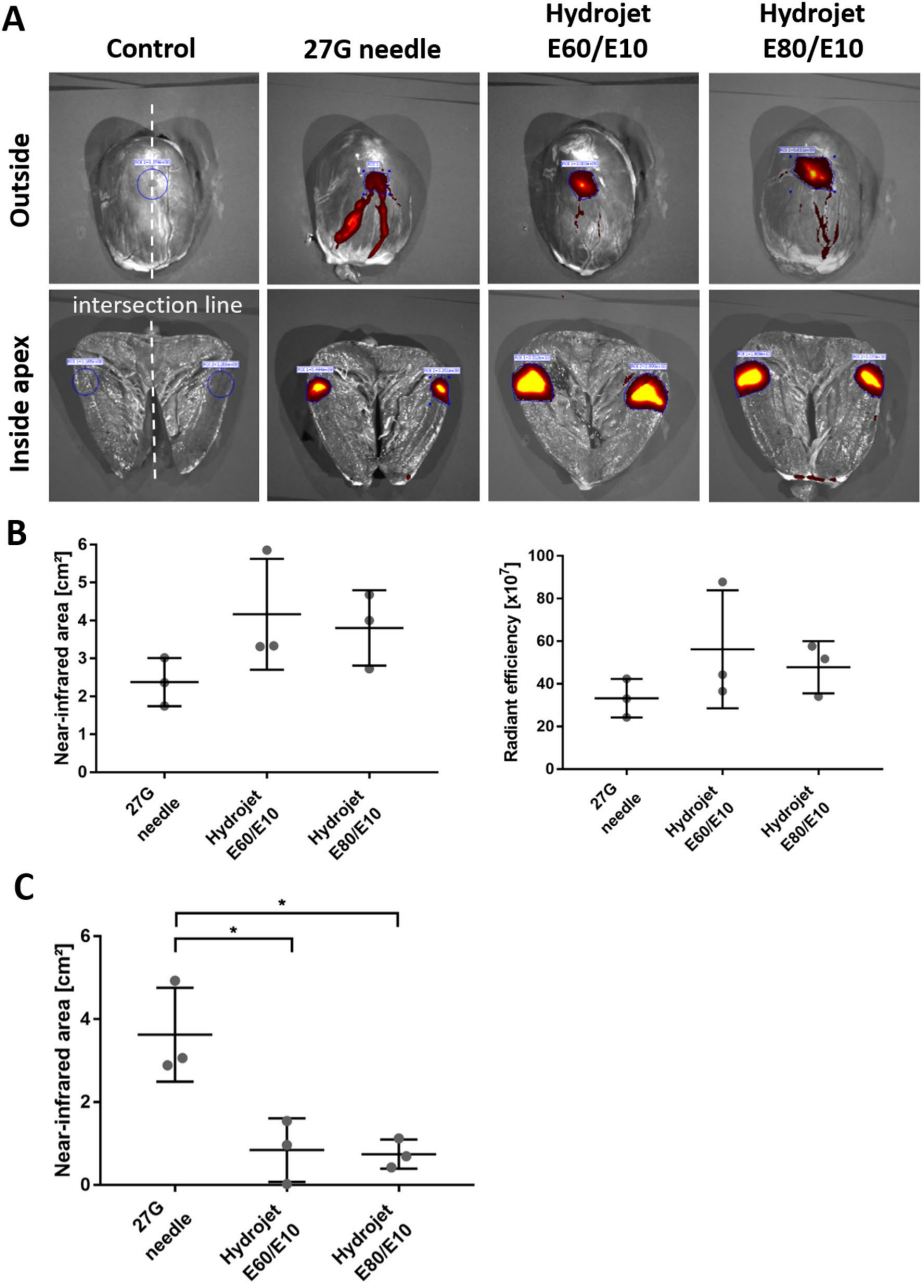
Distribution of cardiomyocytes in porcine hearts after the application with hydrojet system. 100 µl CMM without or with XenoLight DiR fluorescent dye-labeled 1 × 10^6^ cardiomyocytes was applied using 27G needle or hydrojet system with tissue penetration pressures of E80 or E60 and injection pressure of E10 (E80/E10 or E60/E10) into porcine hearts. (**A)** IVIS images of the apex region of the hearts from outside and inside of the myocardium. The intersection of the apex is schematically indicated as a white line. (**B)** Comparison of the radiant efficiency and near-infrared area after the application of cardiomyocytes into the myocardium. (**C)** Detection of the NIR-labeled area of blood vessels containing cardiomyocytes. Results are shown as mean ± SD (n = 3). Statistical differences were determined using one-way ANOVA followed by Bonferroni’s multiple comparison test (*p < 0.05).

To determine the distribution of cardiomyocytes in porcine hearts, 1 × 10^6^ XenoLight DiR fluorescent dye-labeled cardiomyocytes were injected into the myocardium using a 27G needle or the hydrojet system with E60/E10 or E80/E10 setting. The NIR-labeled cardiomyocytes were detected using IVIS in the apex region of the hearts before and after the cutting of the injection site in two halves for detection of the cells inside the myocardium (Fig. 5A). Inside the myocardium, the needle injection showed the smallest distribution area (2.3 ± 0.6 cm^2^) as well as the lowest radiant efficiency (33.2 ± 9.0 × 10^7^) compared to the application of cardiomyocytes using the hydrojet system (E60/10: distribution area of 4.2 ± 1.5 cm^2^, radiant efficiency of 56.2 × 10^7^ ± 27.6 × 10^7^; E80/10: distribution area of 3.8 ± 1.0 cm^2^, radiant efficiency of 47.8 × 10^7^ ± 12.2 × 10^7^) (Fig. 5B). Interestingly, the needle injection resulted in a significantly more pronounced distribution of cardiomyocytes via blood vessels to undesired regions of the heart (Fig. 5A) compared to the hydrojet injections [27G: 3.6  ± 1.1 cm^2^ , E60/10: 0.8  ± 0.8 cm^2^ (p* = 0.0174), E80/10: 0.7  ± 0.4 cm^2^ (p* = 0.0149)] (Fig. 5C).

## Discussion

Over the past decade, the ability to generate patient-specific iPSCs from somatic cells has led to significant advances in regenerative medicine and tissue engineering, which raise the hope for healing infarcted myocardium. In this study, we evaluated the deliverability of patient-specific cardiomyocytes derived from footprint-free iPSCs using a new hydrojet system into the myocardium and analyzed the distribution of the delivered cells in comparison to standard needle injection. The MRI and IVIS analyses demonstrated that the hydrojet system can be used to transfer cardiomyocytes into the myocardium with an improved distribution and significantly less injury of cardiac blood vessels compared to the single needle injection. The in vitro analyses showed that the transfer of cardiomyocytes by hydrojet with appropriate settings does not impair the recovery rate. The new hydrojet system allowed the precise application of two different jet pressures. The first jet enabled the penetration of the tissue (here epicardium and partly the myocardium) while the second jet gently transferred the cells into the target region (here the myocardium).

The transfer of cardiomyocytes by hydrojet injection pressures of E5 and E10 had no significant influence on the recovery rate when compared to the injection with a 27G needle. In contrast, the transfer of cells at higher pressures, i.e. E20, led to a significant loss of the initial cell numbers. These results were not unexpected, as higher pressures correlate positively with velocity and, accordingly, increased shear stress which facilitates cell disruption^33^. Similar results have recently been shown for the injection of MSCs into the urinary sphincter complex^32^.

Immediately after the injection of cardiomyocytes by 27G needle or hydrojet, no influence on cell viability was detected using trypan blue staining. However, 24 h after the cultivation of these cells, a slightly decreased cell viability was detected in cardiomyocytes injected with hydrojet compared to 27G needle injection. After 7 days of cultivation, the viability of cells applied with E20 pressure setting remained still significantly lower than the needle injection. The viability of cells applied with E5 and E10 pressure settings was not significantly different but lower than the needle application.

Even though single-needle injection is a widespread cell delivery technique^34–37^, needle injections generally bear multifactorial disadvantages that may influence the viability, placement, retention rate, or distribution of cells^31^. In our study, a 27G needle was used as reference, which represents a common needle size since previous cardiomyocyte injection experiments report varying needle sizes from 23 to 29G^34–38^. While small needle sizes can lead to increased damage of the cardiomyocytes due to higher shear stress and pressures generated during the injection, needles with a larger diameter have an increased risk of tissue and blood vessel injury or facilitate the reflux of the cells along the penetration tract^31,39^. Concerning the specific application here, needle injection methods can cause a mechanical injury of healthy myocardial tissue and lead to inflammation of the myocardium, which in turn can increase the risk of cardiac arrhythmia^40^. Moreover, needle injection leads to the injury of cardiac blood vessels and thereby to an undesired spread of cardiomyocytes via blood vessels to untargeted regions in the heart. In vivo, the aggregation of cardiomyocytes in the coronary arteries could result in blockage of vessels and induce ischemia.

Different approaches were applied to deliver cells into the myocardium such as intravenous infusion^41,42^, perfusion via the cardiac arteries^43–47^ or multiple injections into the myocardium^37,48^. In a recent study, Tabei et al. applied a newly developed injection device with six needles to deliver human iPSC-derived cardiomyocyte spheroids into the myocardium^38^. Thereby, a retention rate of approximately 48% was achieved compared to the retention rate of around 17% using a single 23G needle. Multiple injections not only affect the retention rate, but also increase the size of the myocardial area in which the cells are distributed. For example, up to 15 injections were applied to deliver cardiomyocytes into the heart of Macaque monkeys^34, 35^. Several clinical studies have shown that about 16% to 21% of the total mass of the left ventricle was affected immediately after the myocardial infarction^49–51^. Thus, to efficiently regenerate the affected myocardium and to restore the functionality, a wide distribution of the injected cells is essential.

In our study, the 27G needle injection resulted in a limited distribution of cells, which was also shown in studies performed by Tabei et al.^38^. In contrast, the sequential application of two differently pressured fluid jets by the new hydrojet system allowed an improved distribution of iron oxide coated microparticles and cardiomyocytes compared to the single-needle injection, without injury of adjacent blood vessels. Thus, the entire infarct area could be covered without major tissue injury by only 2 to 3 repeated injections with the hydrojet. However, further in vivo studies with larger cohort sizes are necessary to establish the exact settings for hydrojet injection into the myocardium. In this study, a trend towards a somewhat more widespread distribution of cardiomyocytes was observed with E60 setting for penetration jet, however, due to the limited sample size, a clear difference between E60 or E80 setting for tissue penetration and cell deposition was not observed.

Both hydrojet application and needle injection were performed epicardially, which is the method applied most frequently for targeted and precise delivery of cells into the infarcted myocardium^52^. This is typically performed under cardiac arrest by open heart thoracotomies^53^ or without cardiac arrest via lateral minithoracotomies^54^. These invasive procedures are associated with a considerable risk of complications. Thus, less invasive catheter-based intramyocardial^55–59^ or intracoronary delivery methods^43–47^ have been already investigated. Injection studies by Grossmann et al. showed an equal or improved distribution when using endocardial applications compared to epicardial administration^60^. Both endocardial and intracoronary administrations are suitable for the new hydrojet system and could make hydrojet-based cell transplantation more precise, less invasive and less traumatic for the patients in the future.

## Conclusion

The novel hydrojet-based cell transfer technology enabled the efficient ex vivo administration of cardiomyocytes into the porcine myocardium using a novel sequential fluid application. Compared to standard needle injections, the hydrojet-based application resulted in significantly less displacement of cells via coronary vessels. Thereby, potential risks due to occlusion of the vessels by aggregated cardiomyocytes and ischemia can be prevented. In the future, this novel cell delivery technique may simplify the treatment of myocardial infarction with patient-specific iPSC-derived cells and increase treatment efficiency.
